# In-stent restenosis and longitudinal stent deformation: a case report

**DOI:** 10.1186/s12872-020-01335-1

**Published:** 2020-01-17

**Authors:** Daoyuan Si, Yaliang Tong, Bo Yu, Yuquan He, Guohui Liu

**Affiliations:** 1 Department of Cardiology, China-Japan Union Hospital of Jilin University, Changchun, Jilin China; 2 Jilin Provincial Engineering Laboratory for Endothelial Function and Genetic Diagnosis of Cardiovascular Disease, Changchun, Jilin China; 3 Jilin Provincial Cardiovascular Research Institute, Xiantai Street NO.126, Changchun, 130033 Jilin China

**Keywords:** In-stent restenosis, Longitudinal stent deformation, Optical coherence tomography, Complication

## Abstract

**Background:**

Longitudinal stent deformation (LSD) is an infrequent complication of percutaneous coronary intervention (PCI), and it may lead to catastrophic clinical outcomes. However, reports of cardiac adverse events associated with LSD are rare.

**Case presentation:**

A 55-year-old man with chest pain was treated for a severe left anterior descending branch (LAD)-diagonal 1 (D1) bifurcation lesion by PCI with two stents in the proximal LAD. LSD occurred during the withdrawal of the trapped D1 wire. High-pressure balloon dilatation was performed in the deformed stent, and the end-angiographic appearance was acceptable, but no additional corrective measures were implemented. Ten months later, the patient represented with acute coronary syndrome. Severe in-stent restenosis (ISR) had suboccluded the proximal LAD, and optical coherence tomography (OCT) visualized multilayered stent struts protruding into the lumen at the compressed segment of the stent. Following complete apposition with balloon dilation, a drug-coated balloon (DCB) was used to avoid an additional permanent metallic layer. He remained angina free, and the angiographic result demonstrated no residual stenosis at the six-month follow-up. To our knowledge, this case demonstrates the first report of ISR triggered by LSD in patients treated with DCBs under the guidance of OCT.

**Conclusions:**

The current report underscores the importance of awareness of LSD, and OCT seems to be the preferred modality to confirm complete apposition. If left without performing additional corrective measures, LSD may be associated with a risk of ISR. Complete apposition with balloon dilation followed by a DCB is a feasible treatment option.

## Background

Longitudinal stent deformation (LSD) has gained more attention recently. To date, the clinical implications of LSD have been uncertain [1–3]. Theoretically, LSD could result in metal overload, malapposition, incomplete plaque coverage and reduced drug delivery, which may lead to a higher risk of stent thrombosis and in-stent restenosis (ISR) [2, 4]. However, reports of adverse events associated with LSD are rare. Here, we present a case of ISR triggered by LSD to raise awareness of this complication. In addition, we also present a treatment using a drug-coated balloon (DCB) under the guidance of optical coherence tomography (OCT).

## Case presentation

**Fig. 1:**
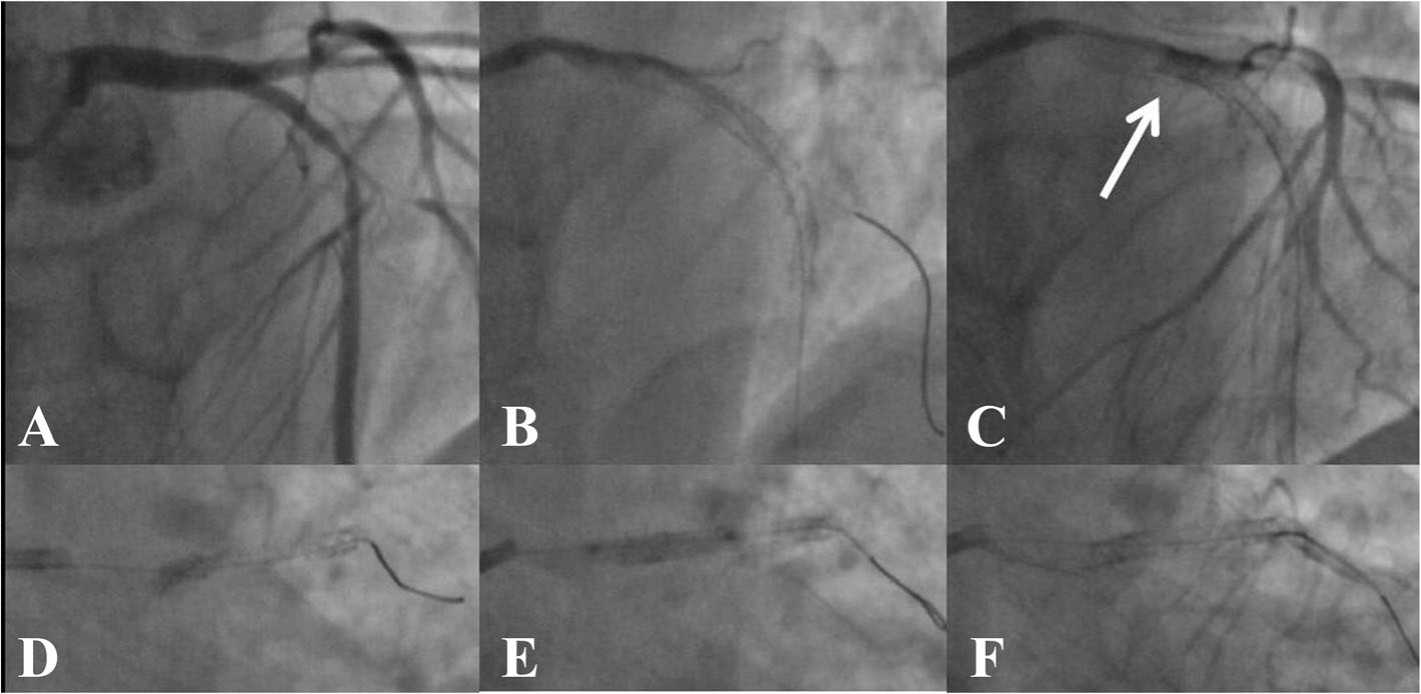
First intervention. **a** Baseline coronary angiogram of the left anterior descending artery (LAD). **b** Two stents were well deployed in the proximal LAD. **c** and **d** Longitudinal compression (white arrow) after removing the trapped D1 guidewire. **e** Postdilatation in the deformed stent. **f** The last coronary angiogram of LAD in the first intervention

A 55-year-old man with a 3-year history of chest pain but no history of myocardial infarction or any intervention was admitted to our hospital, after experiencing exacerbation of pain for 5 days. The electrocardiogram (ECG) showed ST-segment depression in lead V2-V5 < 0.1 mV and transthoracic echocardiogram (TTE) revealed left anterior wall hypokinesis. The physical exam and the laboratory findings including cardiac troponin I were unremarkable. The selective coronary angiography revealed a severe bifurcation lesion of Medina type 1,1,1 involving the left anterior descending branch (LAD) and diagonal 1 (D1) (Fig. 1a). Cardiac catheterization was then performed via the right radial artery using a 6 Fr EBU 3.5 guiding catheter (Medtronic, USA). Both LAD and D1 lesions were individually crossed with Balance Middleweight guidewires (Abbott, USA) and sequentially predilated with 2.0 × 15 mm semicompliant balloons (Boston Scientific, USA). Two Element stents (2.75 × 24 mm distally and 3.0 × 16 mm proximally, Boston Scientific, USA) were deployed in the proximal LAD with the crossover technique (Fig. 1b). Both stents were postdilated at high pressure with a 3.0 × 15 mm noncompliant (NC) balloon (Abbott, USA). Despite careful withdrawal of the trapped D1 wire, a deep engagement of the guiding catheter occurred. It was then noted that the proximal edge of the stent exhibited features of longitudinal compression (Fig. 1c and d). Following postdilatation in the deformed stent using a 3.0 × 15 mm TREK NC balloon (Abbott, USA, Fig. 1e), the end-angiographic appearance was acceptable (Fig. 1f). The patient was treated with 75 mg of clopidogrel for 1.5 years and 100 mg of aspirin lifelong.

**Fig. 2:**
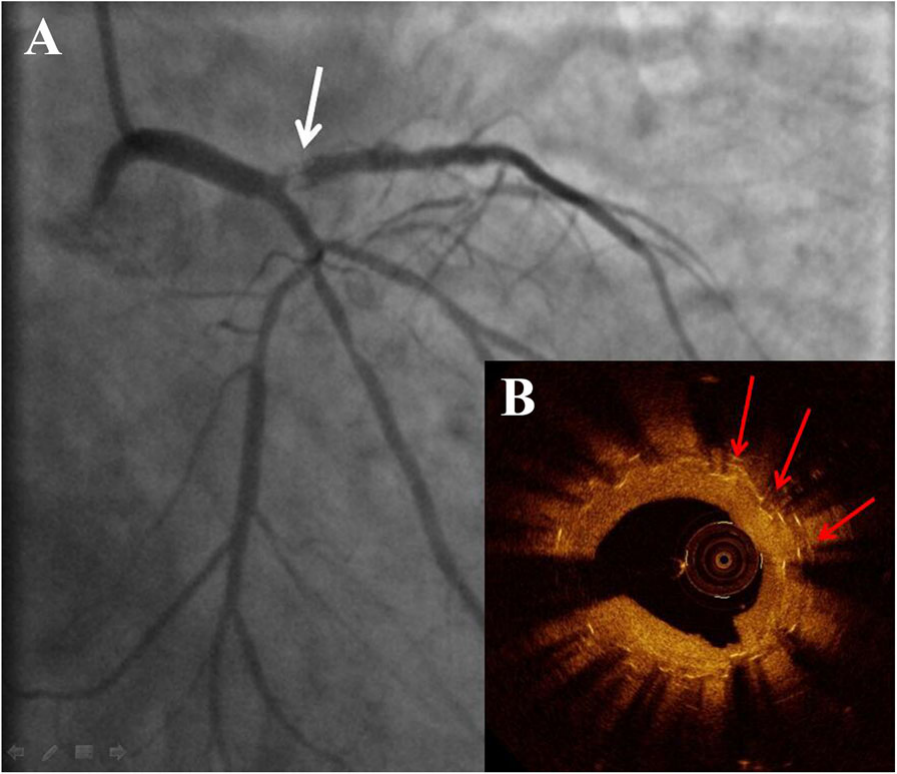
Second angiography. **a** The coronary angiogram showing in-stent stenosis in the proximal left anterior descending artery (white arrow). **b** Optical coherence tomography revealing that the ostial segment of the stent was compressed longitudinally (red arrows indicating multiple strut layers)

**Fig. 3:**
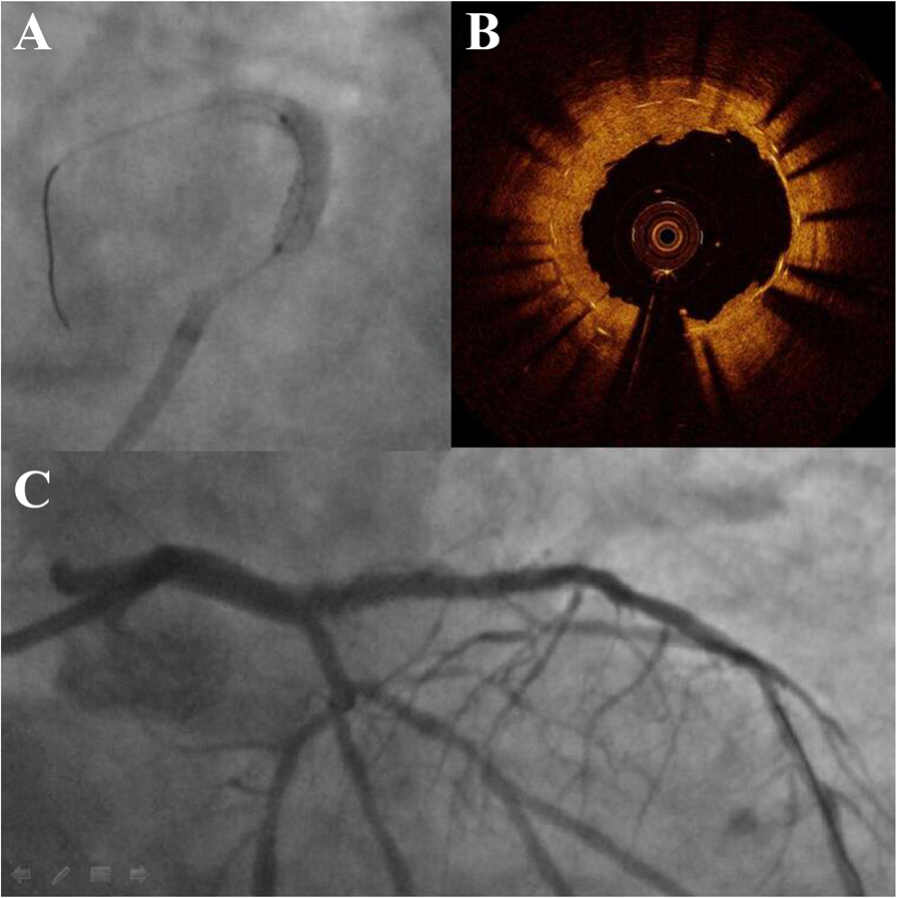
Final result. **a** Treatment with the drug-eluting balloon. **b** Optical coherence tomography showing reduced strut accumulation within the dilated stent lumen. **c** The final coronary angiogram of the left coronary artery showing no residual stenosis

Ten months later, the patient represented with acute coronary syndrome. The ECG showed ST-segment depression in lead V2-V5 = 0.2 mV and the cardiac troponin I was 0.23 ng/ml. The urgent coronary angiography showed a suboccluded proximal LAD due to the severe ISR (Fig. 2a). Following gentle wiring and predilatation, optical coherence tomography (OCT, St. Jude Medical, USA) showed that the proximal segment of the stent was longitudinally compressed and that there were multilayered stent struts protruding into the lumen (Fig. 2b). The joint decision of our cardiology team was in favor of a drug-coated balloon. Thus, the postdilatation was performed with a 3.0 × 12 mm NC balloon (Yinyi, China), followed by a 3.0 × 26 mm SeQuent Please paclitaxel-eluting balloon (B. Braun Melsungen, Germany) at 16 atm with sustained insufflation for 30 s (Fig. 3a). With the excellent angiographic result (Fig. 3c), subsequent OCT imaging showed the improved apposition of the compressed segment and the reduced strut accumulation (Fig. 3b). The procedure was uneventful, and the patient was discharged after a few days. At the six-month follow-up, he remained angina free, and the angiographic results demonstrated no residual stenosis.

## Discussion and conclusion

Although longitudinal stent deformation is still a rare phenomenon, some studies have emphasized the increased rate of LSD in the last few years [2, 4]. The reported cases include all kinds of stent designs, but the Promus Element stent has a relatively high occurrence. Its design with thin struts and fewer connectors are believed to be prone to LSD. On the other hand, its relatively high radio-opacity also provides easier recognition of LSD [1, 5, 6]. The Promus Element stent was also used in the case reported here. The lesion and procedural characteristics are also considered to play a role in the etiology of LSD. Almost all reports cases generally stated that LSD was more frequently observed in calcified lesions, tortuous lesions, ostial and bifurcation lesions. Moreover, its occurrence was also related to some procedural characteristics, such as aggressive guide catheter manipulation, multiple balloons, bifurcation stent techniques, rotational atherectomy, and OCT techniques [2, 7–9]. In a recent study composed of 750 lesions in 5871 patients, Rhee et al. [1] reported that provisional side-branch stenting or ballooning, additional downstream intervention and IVUS use were independently associated with LSD. In our case, deformation was due to guided catheter compression of the ostial segment of the proximal stent after withdrawing the jailed wire in the D1 branch, and post dilatation before removing the jaled wire might make it worse. Therefore, aggressive catheter manipulation for proximal lesions should be avoided to prevent LSD.

To date, the long-term clinical outcomes due to LSD have been uncertain because of the rarity and variability of LSD. Although some recent studies have shown that LSD has no effect on major adverse cardiac events [8, 10], most previous studies and cases have reported that the identification of LSD is important because it may be associated with mechanical and clinical risks, including underexpansion, malapposition, stent thrombosis (ST) and in-stent restenosis (ISR) [3, 4, 11, 12]. Although rare, ST is a severe complication with a mortality rate of up to 45% and a high relapse rate [13]. Mamas et al. [4] reported that even with successful identification and treatment, LSD might lead to a risk of subsequent stent thrombosis. Similar to that case, LSD was identified and treated appropriately at the first percutaneous coronary intervention (PCI) in our case, but ISR occurred at the LSD segment 10 months later. An explanation for this phenomenon might be that although the apposition was rendered with balloon postdilatation and the angiographic appearance was acceptable at the end of the first PCI, incomplete plaque coverage or stent malapposition, which was difficult to detect by sole angiographic evaluation, could have led to the subsequent ISR. Therefore, when LSD is suspected, IVUS or OCT should be used to confirm full lesion coverage and stent apposition to minimize future cardiac adverse events [14]. Owing to its high resolution, OCT may be better at detailed images of the strut distribution and the lumen interface.

Treating patients with ISR is still challenging. The current guidelines on myocardial revascularization recommend restenting with drug-eluting stents (DESs) and the use of DCBs [15]. In our case, the overlap of multiple stent layers was detected by OCT, so restenting with DES might raise concerns about the addition of a permanent metallic layer. The multiple layers may promote further endothelial growth associated with mechanical complications, including stent thrombosis and recurrent ISR [16]. Following complete apposition with balloon dilation, a DCB was used to avoid the additional permanent metallic layer. To our knowledge, this case demonstrates the first report of ISR triggered by LSD in patients treated with DCBs.

LSD should be treated at the time of the initial stent implantation procedure, and high-resolution OCT seems the preferred modality to confirm the complete apposition. If left without performing additional corrective measures, LSD may be associated with a risk of ISR. Despite second-generation DESs and DCBs, both are currently recommended for ISR treatment, but DCBs might be a better option for ISR related to LSD.

## Data Availability

All relevant data is contained within the manuscript.

## Abbreviations

D1: Diagonal-1
DCB: Drug-coated balloon
DES: Drug-eluting stents
ISR: In-stent restenosis
ISR: In-stent restenosis
IVUS: Intravascular ultrasound
LAD: Left anterior descending
LSD: Longitudinal stent deformation
NC: Noncompliant
OCT: Optical coherence tomography
PCI: Percutaneous coronary intervention
ST: Stent thrombosis
